# Missing Insight Into T and B Cell Responses in Dermatitis Herpetiformis

**DOI:** 10.3389/fimmu.2021.657280

**Published:** 2021-03-29

**Authors:** Esko Kemppainen, Teea Salmi, Katri Lindfors

**Affiliations:** ^1^ Celiac Disease Research Center, Faculty of Medicine and Health Technology, Tampere University, Tampere, Finland; ^2^ Department of Dermatology, Tampere University Hospital, Tampere, Finland

**Keywords:** dermatitis herpetiformis, celiac disease, T cell, B cell, epitope spreading, transglutaminase

## Abstract

Dermatitis herpetiformis is a cutaneous form of celiac disease manifesting as an itching rash typically on the elbows, knees and buttocks. It is driven by the ingestion of gluten-containing cereals and characterized by granular deposits of immunoglobulin A in the papillary dermis. These antibodies target transglutaminase (TG) 3 and in the majority of patients they are also found in circulation. The circulating antibodies disappear and skin symptoms resolve as a result of gluten-free diet but the cutaneous anti-TG3 IgA deposits may persist for several years. In dermatitis herpetiformis, plasma cells secreting antibodies against TG3 are located in the intestinal mucosa similarly to those producing TG2 antibodies characteristic for celiac disease. In fact, both TG2- and TG3-specific plasma cells and gluten responsive T cells are found in dermatitis herpetiformis patients but the interplay between these cell populations is unknown. The small bowel mucosal damage in celiac disease is believed to be mediated by co-operation of cytotoxic intraepithelial T cells and the inflammatory milieu contributed by gluten-reactive CD4+ T cells, whereas the skin lesions in dermatitis herpetiformis appear to be devoid of gluten reactive T cells. Thus, how celiac disease-type intestinal T and B cell responses develop into an autoimmune condition affecting the skin is still incompletely understood. Finally, the skin and small bowel lesions may reappear upon reintroduction of gluten in patients treated with gluten-free diet but virtually nothing is known about the long-lived B cell and memory T cell populations activating in response to dietary gluten in dermatitis herpetiformis.

## Introduction

Dermatitis herpetiformis (DH) is a cutaneous form of celiac disease (CeD) usually presenting as a blistering, itching rash particularly on the elbows, knees and buttocks. Both manifestations are driven by the ingestion of dietary gluten in wheat, rye and barley, which induces an inflammatory response featured by B and T cell activation. DH is characterized by granular deposits of immunoglobulin A (IgA) in the papillary dermis, which is considered the primary diagnostic criterion for the disease (1). These antibodies target the human transglutaminase (TG) 3 and are also found in the circulation of the majority of DH patients (2, 3). The circulating antibodies disappear, and skin symptoms resolve on a gluten-free diet (GFD), the treatment of choice in DH, while the anti-TG3 IgA deposits in the skin may persist for several years or even decades despite dietary adherence (2, 4, 5).

Regardless of the differing primary manifestations, DH and CeD share genetic susceptibility conferred by HLA-DQ2 or -DQ8 (6) and present often with partially overlapping features (**Table 1**). The great majority of untreated CeD patients are seropositive for gluten-dependent antibodies against gluten-derived gliadin peptides and autoantibodies against TG2 (anti-TG2 and endomysial antibodies), another member of the TG family of enzymes and the main autoantigen in CeD (7). Likewise, most DH patients are seropositive for TG2 autoantibodies (25). Moreover, approximately 30% of the CeD patients are also seropositive for TG3-autoantibodies while the corresponding number among DH patients has been shown to be considerably higher, ranging from 52-95% in reported studies (3, 16, 17). In addition, the vast majority of DH patients exhibit small-bowel mucosal tissue remodeling and damage, *i.e.* villous atrophy, crypt hyperplasia and inflammation, characteristic of CeD (26, 27). In this review, DH in patients either with or without villous atrophy was compared to CeD patients with no DH rash.

**Table 1:** Comparison of the features of dermatitis herpetiformis and celiac disease.

Feature	Dermatitis herpetiformis	Celiac disease	Reference
**Primary autoantigen**	Transglutaminase 3	Transglutaminase 2	(2, 7)
**Dermal TG3-IgA deposits**	100%	Not determined*	(8, 9)
**Villous atrophy**	Approx. 75%	Nearly 100%	(10)
**CD3+/γδ+ IELs**	70%/91% of patients	93%/93% of patients	(11, 12)
**Intestinal TG2-IgA**	79%	100%	(12, 13)
**Intestinal plasma cells**	Anti-TG2: detected Anti-TG3: detected	Anti-TG2: detected Anti-TG3: rarely detected	(14, 15)
**Serum TG3-IgA**	52-95% of patients	33-53% of patients	(3, 16–18)
**Serum TG2-IgA**	45-95% of patients	Nearly 100% of patients	(3, 17, 18)
**Serum DGP-IgA/IgG**	78%/78%	74%/65%	(19, 20)
**HLA-DQ2/DQ8**	86-100%/0-12%	88-95%/4-6%	(6, 21, 22)
**T cell response**	Not characterized	T_H_1	(23, 24)

*IgA and TG3 deposits detected in some celiac disease patients (8).

## B Cell Responses in DH

Both DH and CeD are characterized by the occurrence of circulating TG2, gliadin and deamidated gliadin peptide antibodies (28). Despite this, the DH specific antibody response is considered to be targeted against the main autoantigen TG3 (2). However, circulating TG3-antibodies are found also in a subset of CeD patients without DH, but their significance in CeD is poorly understood. TG2, the main autoantigen in CeD, can both deamidate gluten peptides and form both iso-peptide linked and thioester-linked complexes with gluten, which are believed to drive TG2 autoantibody production in CeD (29). TG3 only forms enzyme-peptide complexes with lower affinity *via* thioester linkage (30). In addition, TG3 is also able to incorporate significantly fewer peptides per enzyme than TG2 (30). The differing end products resulting from TG2 or TG3-catalyzed reactions may explain the different dynamics of the autoantibody responses in CeD and DH. Furthermore, the complement of immunogenic gluten-derived peptides, which can act as TG3 substrates, the complement of T cell receptor subsets, and their impact on the development of B cell mediated immune response in DH have not been studied.

In CeD, TG2-antibody producing plasma cells are found in the small intestinal lamina propria (31, 32), although circulating antibodies may originate outside the intestine despite strong clonal relatedness between circulating and gut-derived autoantibodies (33). Intestinal plasma cells producing autoantibodies against TG2 have also been discovered in DH patients (14). Recently, the first studies on the occurrence of TG3 autoantibody producing cells in DH were published. *Ex vivo* cultures of duodenal biopsies as well as intestinal plasma cell stainings performed on DH patient tissue strongly suggest that TG3-antibody producing cells are present at least in the small intestine (14, 34). These cells seem to be highly DH-specific: despite the occasional occurrence of circulating TG3 antibodies, TG3 antibody producing plasma cells have only rarely been detected in CeD patients (14, 34). Furthermore, the TG3-specific plasma cells appear to be gluten-responsive as their frequency is increased during gluten challenge (14). However, according to current evidence, the presence of intestinal anti-TG3 plasma cells seems not to consistently correlate with the level of serum TG3 antibodies (14), raising the possibility that two or more subsets of autoantibodies with different plasma cell origins may exist in DH, as suggested for TG2 autoantibody producing plasma cells in CeD (33).

Supporting the hypothesis of a strictly DH-specific TG3 autoantibody plasma cell subpopulation, the number of intestinal TG3 autoantibody producing plasma cells detected in DH patients’ gut biopsies using biotinylated TG3 to visualize TG3-specific antibody producing cells was not affected by preincubation with recombinantly produced TG2 (33). This suggests that these cells have a high specificity to TG3 alone. Likewise, CeD patients’ recombinant monoclonal TG2 intestinal antibodies have been demonstrated to lack cross-reactivity with TG3 (35). Despite these findings implying very strict epitope specificities, it has been suggested that the multiple co-existing antibody populations would arise through epitope spreading, *i.e*. initial autoimmunity against TG2 would later expand to cover other closely related members of the same transglutaminase family (36). This hypothesis was supported by the pivotal work of Sárdy and colleagues (2) suggesting the possible existence of two distinct populations of TG3-antibodies: one highly specific to TG3 and present only in DH patients and the other recognizing both TG2 and TG3 and potentially present in both DH patients and CeD patients without DH. While the epitope spreading hypothesis remains plausible, testing it would first require identifying the specific TG3 epitopes recognized by the DH patient antibodies.

## Systemic T Cell Responses in DH

**Figure 1:**
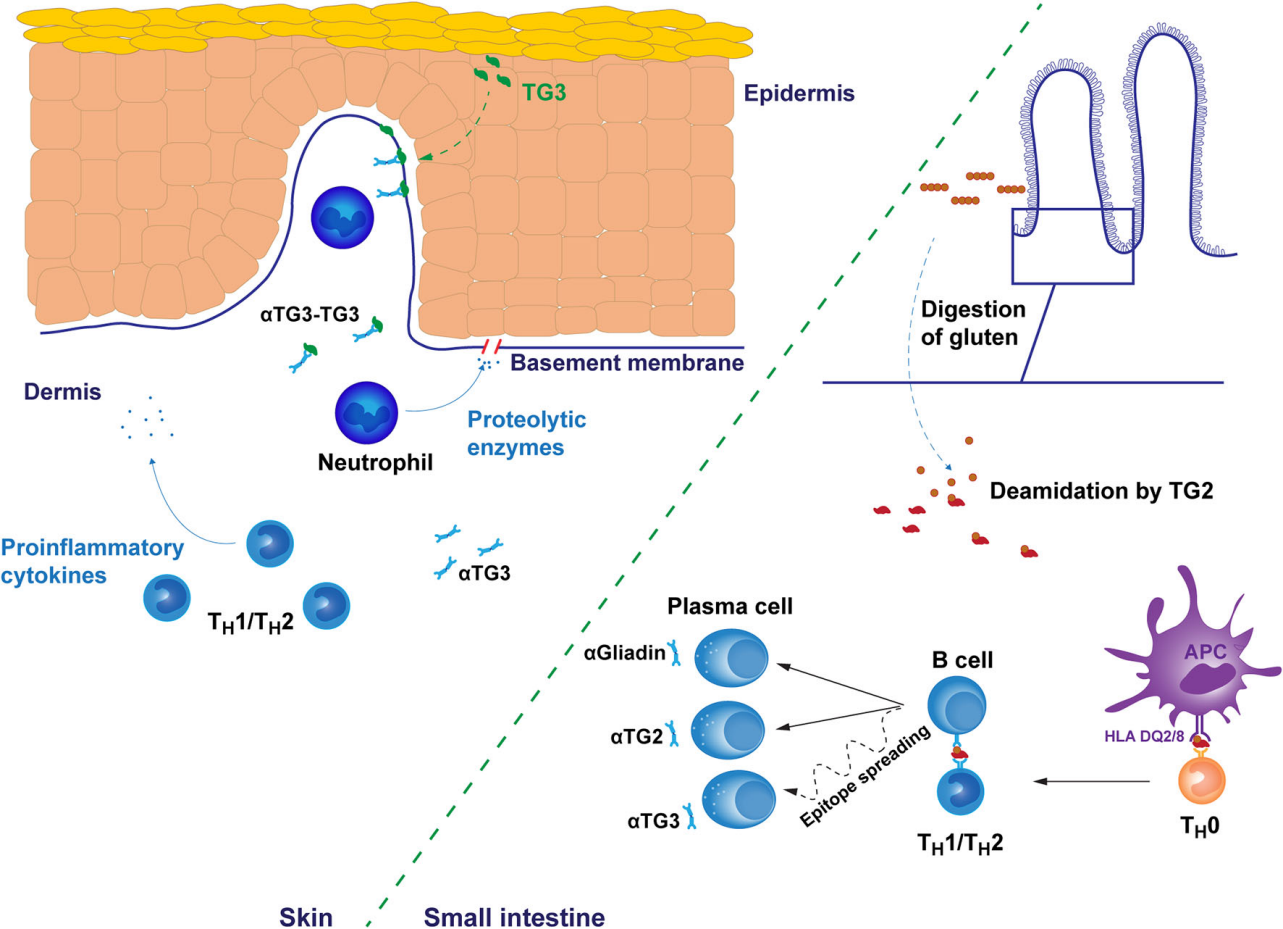
Putative pathogenetic mechanisms of dermatitis herpetiformis. The early gluten-induced autoimmune response results in the production of autoantibodies targeting gluten-derived gliadin peptides and transglutaminase 2 (TG2) in the small bowel mucosa. Antibody specificity to transglutaminase 3 (TG3) may develop later by epitope spreading after initial autoreactivity against TG2. However, the dynamics of this processes are unknown. Immune responses are mediated by subtype 1 or 2 T helper cells. In the skin, IgA and TG3 form punctate structures, typically found in the dermal papillae. TG3 is endogenously produced in the upper layers of epidermis but may spontaneously diffuse towards dermis providing a possible explanation for the recruitment of autoantibodies into tissue bound immunocomplexes. Alternatively, IgA-TG3 immunocomplexes circulating free in dermatitis herpetiformis patient serum may bind to these sites. Tissue bound immunocomplexes attract neutrophil infiltration to dermal-epidermal junctions resulting in the cleavage of the basement membrane and finally in blister formation. The membrane cleavage is likely catalyzed by neutrophil secreted proteolytic enzymes targeting the fibrillar proteins of the basement membrane. αTG2/3, TG2/3 autoantibody, αGliadin, anti-gliadin antibody, APC, antigen presenting cell.

The disease pathogenesis of both CeD and DH is considered to involve a major T cell component. In CeD, the gluten reactivity of intestinal T cells is strongly associated with the DQ2.5 molecule and the crosstalk between B cells and T cells reacting to covalently linked peptide-TG2 complexes is key in the generation of the TG2 autoantibody response. However, the actual intestinal epithelial cell destruction in CeD is mediated by intraepithelial cytotoxic CD8 T lymphocytes (23). The cascade of T and B cell driven events induced by the ingestion of gluten, is far less well characterized, however, in the case of DH although the early events occurring in the small intestine are thought to follow the same path in both manifestations (**Figure 1**.).

Only few studies have assessed the immune cell subsets and levels of T cell derived cytokines in DH. The peripheral T cell responses to gluten in GFD-treated DH and CeD patients following a gluten-challenge were assessed in a recent study (37). DH patient-derived peripheral blood mononuclear cells (PBMCs) responded to two CeD-associated gliadin peptides (DQ2-glia-α1a/α2 and DQ2-glia-ω1/ω2 peptides) in an interferon-γ ELISpot (enzyme-linked immunospot assay) qualitatively similarly to those obtained from CeD patients. It is noteworthy, however, that while CeD is considered to be a strictly Th1-mediated disease (38), earlier studies suggest that this may not be the case with DH, *i.e.* the proportion of interferon-γ-secreting T cells among circulating immune cells might thus be low in DH patients. Th2-related cytokines in turn, such as interleukin (IL)-4 and IL-5 have been found to be overexpressed both in the skin and in the serum of patients with DH (24, 39). The exact nature of the gluten-induced T cell response thus remains to be ascertained.

## Skin Lesions Are Devoid of Gluten-Reactive T Cells

The mechanisms underlying the skin lesions in DH are only superficially understood. The pathognomonic granular deposits of IgA co-localize with TG3 in the papillary dermis, and are located particularly in perilesional areas of the skin (2, 40, 41). TG3 is not endogenously produced by the cells lining the dermal-epidermal boundary and it is perplexing why TG3 and the autoantibodies precipitate to such persistent punctate structures at these loci. Hypothetically, the TG3-IgA immunocomplexes found in the circulation of DH patients (42) may simply adhere to structural proteins, for instance fibrinogen (40), potentially substrates for TG3, of the dermal papillae. Alternatively, TG3 may diffuse from the epidermis to the dermis, where it would be bound by circulating TG3 antibodies (43). Moreover, it is unclear why dermal TG3-IgA deposits may persist for years despite strict adherence to GFD and faster disappearance of detectable levels of circulating TG3-antibodies (1, 5). Prolonged clearance of the deposits is one possible explanation. However, the existence of small, persistent populations of antibody-producing cells in lymphatic tissue cannot be excluded since virtually nothing is known about the possible development of long-lived plasma cells or memory T cells in DH. For example, such long-lived TG2 antibody producing plasma cells populations discovered in CeD patients (44, 45) have not been investigated in DH.

It is noteworthy that the presence of the tissue bound TG3-IgA immunocomplexes alone appears not to be pathogenic, since these deposits are at times also detectable in skin areas of DH patients far away from the rash and also in the skin of DH patients in clinical remission (5, 46). Also, a few studies have presented granular IgA deposits-findings also in CeD patients without DH (8, 47, 48), but to our knowledge in only one study IgA was shown to co-localize with TG3 (8). Some of the early studies on the disease pathogenesis suggest that the formation of skin lesions in DH involves an influx of lymphocytes and macrophages (49, 50) but, contrary to the duodenum in CeD, the skin lesions appear to be devoid of gluten-reactive T cells (51). In a murine model of DH, the skin lesions develop virtually in complete absence of local CD4+ T cells, driven mainly by neutrophils and monocytes (52).

Indeed, the affected skin areas in DH have been shown to be infiltrated by neutrophils (53, 54), which have the ability to secrete proteolytic enzymes such as collagenases, elastases and granzyme B. These enzymes may be responsible for the disruption of connective tissue between the dermis and epidermis DH, resulting in blister formation (55). It is also noteworthy that dapsone, a drug that shows rapid clearance of DH rash (10) is a potent anti-neutrophilic agent (56, 57). Smith and colleagues showed that these dermal neutrophils have an increased ability to bind IgA *via* their Fc IgA receptors, indicative of prior priming (54). It is compelling to hypothesize that this neutrophil priming may occur in the inflamed intestine. The dermal immune infiltrate in DH also comprises at least αβ and γδ subtypes of T cells (58, 59). Increased numbers of intraepithelial αβ and γδ T cells in the small bowel mucosa is one of the hallmarks of both DH and CeD (11, 60) but whether the populations in skin and small bowel are linked remains an open and interesting question. The evidence at least for γδ T cells so far would suggest that this is not the case: Holtmeier and colleagues studied the TCR δ repertoires present in the inflamed duodenum, peripheral blood, involved and non-involved skin of DH patients and found that cutaneous TCR δ repertoires were oligoclonal, and that identical dominant γδ T cell clones were present throughout lesional and perilesional skin (61). Furthermore, the TCR δ repertoires of blood, the small intestine and skin were different and thus the cutaneous γδ T cells are not likely to originate from the inflamed duodenum.

The loss of tolerance to gluten and self-antigens may also be caused by impaired regulatory T (Treg) cell compartment. Loss of Treg suppressivity has been linked to CeD (62, 63) and the same impaired function could affect the cutaneous Treg population in DH. The potential role of T regulatory cells in DH pathology is supported by reduced levels of Foxp3+ Treg cells in DH patients’ skin, as reported by Antiga and colleagues (64). This phenomenon has also been reported in other autoimmune disorders of the skin, such as systemic scleroderma (65) and bullous pemphigoid (66).

## Discussion

The understanding of DH pathogenesis has increased significantly in recent decades. The origins of the blistering skin condition are most likely in the inflamed small bowel, but it is unknown, how the autoimmunity progresses from the gut to the skin. Epitope spreading from initial immune response against TG2 to TG3 has been suggested as a possible mechanism (36). This is supported by the fact that DH generally tends to develop later than CeD and that CeD can progress into DH, particularly if dietary adherence is not optimal (67). In addition, CeD and DH patients have also been shown to develop antibodies against another closely related transglutaminase, TG6 (30, 36, 68). It is also noteworthy that such a pathogenic process may be exacerbated by a number of intrinsic and extrinsic factors. For example, ageing is considered detrimental to the functionality of T cell mediated immunity (69), but certain processes such as the impairment of the self/non-self-discrimination and subsequent accumulation of self-reactive memory B cells (70) may also contribute to the development of DH later in life. Furthermore, the impact of accumulating exposure to environmental stressors such as infections or environmental toxins has not been thoroughly studied in the case of DH, although it is known that, for example iodine, exacerbates the skin lesions, potentially by causing aberrant activity of the skin immuno-complex associated TG3 (71). Finally, many of the potential environmental modulators are linked to the intestinal microbiota, and while gut dysbiosis has been tentatively linked to CeD (72), there have been no studies on how the maintenance of the microbial homeostasis throughout life might impact the development of DH.

The early T cell responses in DH in general are far less well understood than in CeD. The complement of gluten-derived immunogenic peptides, gluten-reactive T cell subsets and their receptor subsets in particular have not been thoroughly investigated. A plethora of cytokines, *e.g.* IL-8, IL-36 and IL-17 (73, 74) have been linked to DH, primarily by virtue of positive correlation between serum levels and disease status or gluten exposure. No conclusive evidence has so far been presented for their exact role in the disease pathophysiology, however. Neither have comprehensive systemic cytokine profiling studies been conducted on DH patients, such as that conducted by Goel and co-workers on CeD patients (75). Yet it is curious that CeD is considered a Th1-mediated autoimmune disorder, while Th2-linked cytokines dominate the molecular findings linked to DH. Whether such a profound difference between these two manifestations of the same disease truly exists, and the dynamics of a possible switch between effector cell subsets, could be an interesting novel avenue in DH research.

One distinguishing characteristic of DH is the pathognomonic granular IgA deposits in the dermal papillae of the skin. These IgA deposits are found particularly in non-lesional skin and thus the possibility of them being merely an epiphenomenon cannot be fully excluded, although their absence from the lesions could also be explained by phagocytic processes. With IgA being the predominant Ig class produced by the intestinal plasma cells, it is plausible that IgA antibodies in the dermal granular deposits originate from the gut. The cutaneous antibodies in DH appear to be dimeric (76) and predominantly of the IgA1 subclass (77, 78), the predominant subclass produced in the small intestine (79). Irrespective of their site of origin, the presence of the secretory component in the dermal immunocomplexes (76) implies that their transcytosis into circulation has been mediated by mucosal epithelial cells expressing the polymeric immunoglobulin receptor (80).

Two major questions thus remain unanswered as regards DH etiology: the origin of the cutaneous IgA deposits and their role in the development of the skin lesions. Very little is also known about the relationship between DH and other autoimmune bullous skin diseases. DH is often perceived primarily as an extraintestinal manifestation of CeD but understanding its immunology could be just as relevant for understanding similar autoimmune skin conditions and vice versa. For example, in a Finnish retrospective case-control study, patients with DH were found to have a 22-fold higher risk of developing bullous pemphigoid, another autoimmune blistering skin disease, compared to the only two-fold higher risk of subsequent bullous pemphigoid development among CeD patients (81).

Much of the work in understanding the cellular and molecular pathophysiology of DH dates back to the 1980s and 1990s and many of the questions could be and should be reassessed with modern methodology. A major hindrance in studying DH is recruitment of patients. Contrary to CeD, the incidence of DH is slowly declining with fewer than 10% of CeD patients developing DH (9). This phenomenon is interesting in its own right and most likely due to the increased awareness and improved diagnostics of CeD, resulting in fewer cases of untreated celiac disease developing into DH. Unfortunately, however, this otherwise positive trend also sets limitations for conducting clinical studies and limits access to patient material. Thus, long-term collaboration and careful coordination of research between clinics and research units is necessary. Furthermore, due to the multifaceted nature of DH, combining the expertise of clinicians from different fields of medicine with that of basic researchers is vital.

## Author Contributions

EK and KL conceptualized the manuscript. All authors contributed to the article and approved the submitted version.

## Funding

This work was supported by the Sigrid Juselius Foundation, by the Academy of Finland (grants 314880 and 315755) and by the Competitive Research Funding of Tampere University Hospital (grants 9X051 and 9AA070).

## Conflict of Interest 

The authors declare that the research was conducted in the absence of any commercial or financial relationships that could be construed as a potential conflict of interest.
